# Autoimmune Acquired Factor V Deficiency in a Patient With Pancreatic Cancer Complicated by Cholangitis: A Rare Coagulopathy With a Favorable Course

**DOI:** 10.7759/cureus.87946

**Published:** 2025-07-14

**Authors:** Tsuyoshi Ueda, Koichiro Miyagawa, Tsukasa Nakanishi, Junichi Tsukada, Masaru Harada

**Affiliations:** 1 Third Department of Internal Medicine, University of Occupational and Environmental Health, Kitakyushu, JPN; 2 Department of Hematology, University of Occupational and Environmental Health, Kitakyushu, JPN

**Keywords:** autoimmune acquired factor v deficiency, cholangitis, coagulopathy, cross mixing test, recurrent biliary obstruction

## Abstract

Autoimmune acquired factor V deficiency (AiFVD) is a rare bleeding disorder characterized by the presence of inhibitors against coagulation factor V (factor V). It can manifest with a wide spectrum of clinical severity, from asymptomatic to life-threatening bleeding. Various underlying causes have been reported, including malignancies, autoimmune disorders, infections, and antibiotics. However, the mechanism remains unclear, and diagnostic delays are common due to its rarity. A 78-year-old woman with pancreatic head cancer underwent bile duct stenting for malignant biliary stricture. She subsequently developed cholangitis from stent obstruction, accompanied by coagulopathy. Coagulation studies revealed a prolonged prothrombin time of 68.1 seconds, an activated partial thromboplastin time exceeding 283.7 seconds, and severely reduced FV activity (<3%). A cross-mixing test suggested the presence of an inhibitor, leading to the diagnosis of AiFVD. The coagulopathy resolved following treatment of the underlying cholangitis. AiFVD can arise from various causes; in this case, it was attributed to cholangitis caused by recurrent biliary obstruction.

## Introduction

Autoimmune coagulation factor deficiency includes acquired hemophilia A (AHA), autoimmune acquired factor V deficiency (AiFVD), autoimmune factor XIII deficiency (AiFXIIID), von Willebrand disease, and autoimmune factor X deficiency [1,2].

Factor V (FV) acts as a cofactor in the prothrombinase complex, playing a critical role in converting prothrombin to thrombin and thereby facilitating fibrin clot formation. When FV is deficient, it can disrupt this process and lead to prolonged bleeding, resulting in impaired hemostasis. AiFVD develops when FV activity is reduced due to the production of FV inhibitors [3]. The incidence of AiFVD has been reported to range from approximately 0.038 to 0.29 per million persons, which is lower than the incidence of AHA [2,4,5].

Acquired FV inhibitors are induced by various conditions, including drug reactions, infections, autoimmune diseases, blood transfusions, pregnancy, malignant diseases, and sepsis [6]. The clinical presentation of AiFVD is heterogeneous and can lead to fatal hemorrhage, although some cases are asymptomatic and resolve spontaneously [5]. Due to its variable symptoms, many cases may remain undiagnosed. We report a case of AiFVD that developed secondary to cholangitis caused by bile duct stent obstruction, which resolved with treatment of the cholangitis alone.

## Case presentation

A 78-year-old woman presented with cholangitis associated with recurrent biliary obstruction (RBO).

Medical history

**Figure 1:**
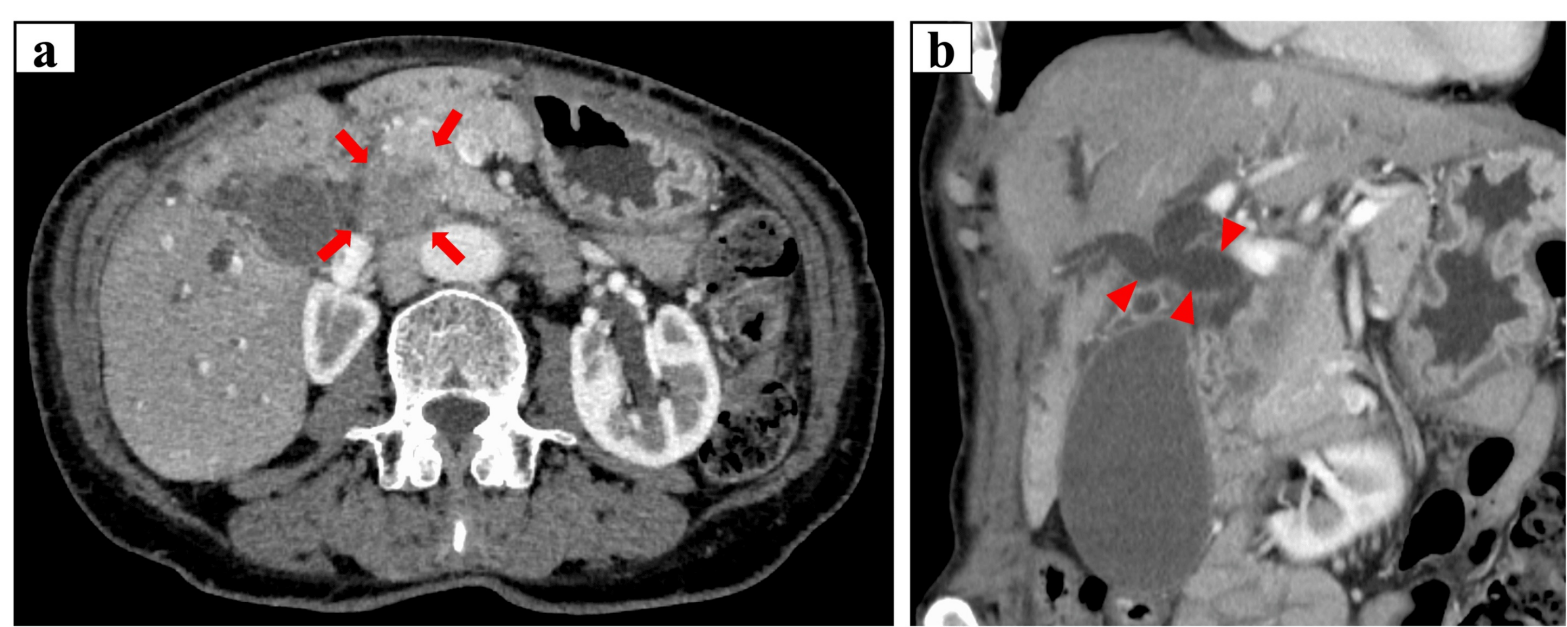
Contrast-enhanced abdominal CT at the initial diagnosis showing (a) a mass located in the head of the pancreas (arrows), and (b) moderate dilation of the common bile duct and intrahepatic bile ducts (arrow heads).

**Figure 2:**
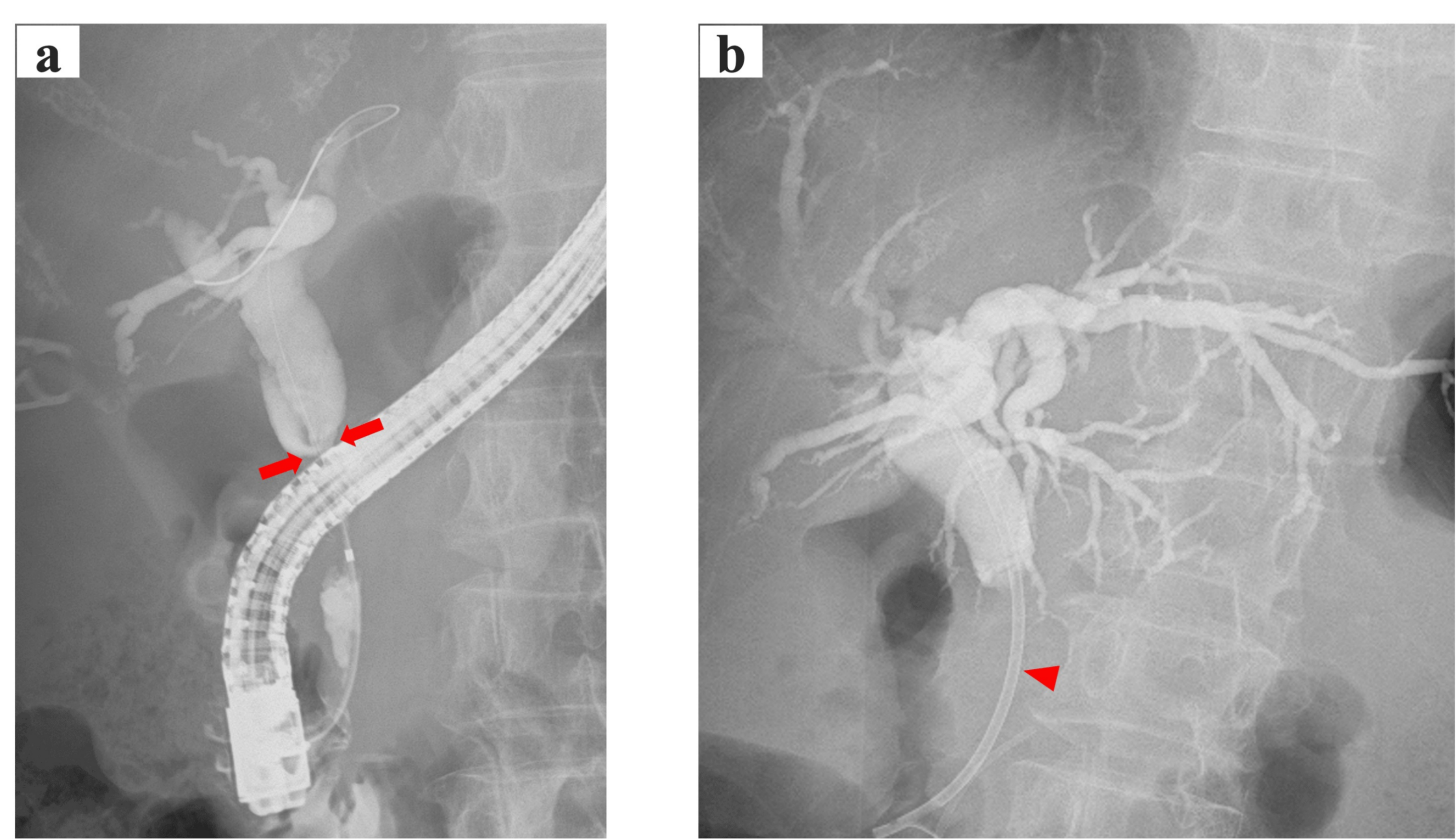
Endoscopic retrograde cholangiopancreatography (ERCP) showing (a) a distal bile duct stricture (arrows), and (b) a plastic stent being placed within the common bile duct (arrow head).

One year prior to presentation, the patient was diagnosed with pancreatic head cancer complicated by obstructive jaundice. At the time of diagnosis, she had no significant medical or family history, including coagulopathy, and her coagulation tests were normal. Abdominal contrast-enhanced computed tomography (CT) revealed intrahepatic bile duct dilation and a 22 mm hypovascular mass in the pancreatic head (Figure 1). Endoscopic retrograde cholangiopancreatography (ERCP) was performed to treat obstructive jaundice and revealed distal bile duct stenosis (Figure 2). Endoscopic ultrasound-guided tissue acquisition (EUS-TA) was performed for pathological diagnosis, confirming the diagnosis of adenocarcinoma. A bile duct stent was placed, and cholestatic parameters improved within a few days following the procedure. The serum carbohydrate antigen (CA) 19-9 level was 78.8 U/mL (normal range < 37.0 U/mL). These findings led to the diagnosis of pancreatic head cancer, Stage II B (according to the American Joint Committee on Cancer classification), and surgical treatment was considered appropriate.

Neoadjuvant chemotherapy (NAC) with gemcitabine and S-1 combination therapy (GS therapy) was initiated. After two cycles of GS therapy, an abdominal CT scan revealed multiple new mass lesions in the liver. Consequently, the treatment regimen was switched to gemcitabine and nab-paclitaxel therapy (GnP therapy) for unresectable pancreatic cancer.

Current presentation

Two months after the initiation of GnP therapy, she was admitted to our hospital due to cholangitis associated with recurrent biliary obstruction (RBO). In the weeks preceding her admission, she had no infections and medications other than chemotherapy. On admission, there were no obvious bleeding symptoms, but microscopic hematuria was observed. The platelet count was within normal limits, but her coagulation parameters were abnormal. Prothrombin time (PT)-international normalized ratio (INR) was 6.97 (reference range 0.84−1.14), and activated partial thromboplastin time (APTT) was 188.1 seconds (reference range 25.2−40.0) (Table 1). 

**Table 1:** Laboratory test results MCV, mean corpuscular volume; RDW, red cell distribution width; Ret, reticulocyte count; IPF, immature platelet fraction; AST, aspartate aminotransferase; ALT, alanine aminotransferase; ALP, alkaline phosphatase; GGT, gamma-glutamyl transferase; LDH, lactate dehydrogenase; BUN, blood urea nitrogen; HbA1c, glycated hemoglobin; CRP, C-reactive protein; PIVKA-II, protein induced by vitamin K absence or antagonist Ⅱ; PT, prothrombin time; PT-INR, prothrombin time-international normalized ratio; APTT, activated partial thromboplastin time; HBsAg, hepatitis B surface antigen; HBcAb, hepatitis B core antibody; HBV-DNA, Hepatitis B virus DNA; HCVAb, hepatitis C virus antibody

Parameters	Patient Value	Reference range
Peripheral blood		
White-cell count (per μL)	6,300	3,300-8,600
Eosinophil (%)	0.8	0-8.5
Basophil (%)	0.3	0-2.5
Lymphocyte (%)	8.7	16.5-49.5
Monocyte (%)	12.6	2.0-10.0
Neutrophil (%)	77.6	38.0-74.0
Red-cell count (per μL)	2,910,000	3,860,000-4,920,000
Hemoglobin (g/dL)	8.7	11.6-14.8
Hematocrit (%)	27.9	35.1-44.4
MCV (fL)	95.3	83.6-98.2
RDW (%)	18.5	11.6-15.6
Ret (%)	3.0	0.5-2.5
Platelet count (per μL)	476,000	158,000-348,000
IPF (%)	1.6	1.1-6.1
Biochemistry		
Total protein (g/dL)	6.2	6.6-8.1
Albumin (g/dL)	3.0	4.1-5.1
Total bilirubin (mg/dL)	3.5	0.4-1.5
AST (U/L)	39	13-30
ALT (U/L)	40	7-23
ALP (U/L)	830	38-113
GGT (U/L)	483	9-32
LDH (U/L)	454	124-222
BUN (mg/dL)	9.0	8-20
Creatinine (mg/dL)	0.56	0.46-0.79
Glucose (mg/dL)	107	73-109
HbA1c(NGSP) (%)	5.4	4.9-6.0
Sodium (mmol/L)	135	138-145
Potassium (mmol/L)	3.9	3.6-4.8
Chloride (mmol/L)	99	101-108
Serology		
CRP (mg/dL)	6.04	≤ 0.14
Tumor marker		
PIVKA-II (mAU/mL)	13.0	≤ 40.0
Coagulation		
PT (sec)	74.3	11.0-13.4
PT (%)	6.5	≥ 74.0
PT-INR	6.97	0.94-1.15
APTT (sec)	188.1	24.0-39.0
Viral marker		
HBsAg (IU/mL)	negative	
HBcAb (S/CO)	5.9	
HBsAb (mIU/mL)	484.9	
HBV-DNA (Log IU/mL)	negative	
HCVAb ( S/CO)	negative	

Diagnosis and management

Notably, her coagulation parameters were normal one month earlier. We suspected that the coagulopathy was caused by cholangitis due to biliary stent obstruction. ERCP is generally avoided under such severe coagulopathy due to the risk of procedure-related bleeding. However, the patient had a normal platelet count and exhibited no overt bleeding symptoms, and given the clinical deterioration, we prioritized the treatment of cholangitis and performed ERCP. According to the Tokyo Guidelines 2018 (TG18) [7], acute cholangitis is classified into three severity grades based on clinical, laboratory, and imaging criteria. In the present case, the patient was classified as having moderate (Grade II) cholangitis, and endoscopic treatment with ERCP was prioritized accordingly.

The bile duct stent was replaced, and antibiotic therapy with sulbactam/cefoperazone (SBT/CPZ) 1 g twice daily was initiated. On the fourth day of hospitalization, coagulation abnormalities persisted, with PT at 7.2% and APTT at 283.7 seconds. Initially, liver failure and vitamin K deficiency were considered as differential diagnoses; however, abdominal imaging showed no signs of liver atrophy, serum PIVKA-II was within the normal range at 13.0 mAU/mL, and vitamin K administration did not improve the coagulation abnormalities. These findings suggested that liver failure and vitamin K-dependent coagulopathy were unlikely.

Further analysis of the coagulation factors revealed that factor II and factor X were within the reference range due to dilution, whereas FV was significantly decreased (Table 2). Anticardiolipin autoantibodies, anti-β2-microglobulin, and anti-cardiolipin-β2-glycoprotein I complex antibodies were not detected (Table 3). This suggests that antiphospholipid syndrome (APS) was not the underlying cause of coagulation abnormalities in this case.

**Table 2:** Patient values of coagulation factor assays and their normal range

Coagulation factor assay	Day 6 after admission	Day 29 after admission	Reference range
Ⅱ	56	82	70-120%
Ⅴ	3	83	70-140%
Ⅹ	43	76	70-120%

**Table 3:** Laboratory and coagulation data on day 6 of admission. CRP, C-reactive protein; aCL, anti cardiolipin; Ab, antibody; IgM, immunoglobulin M; IgG, immunoglobulin G; β2GPI, β2 glycoprotein-I; PT, prothrombin time; PT-INR, prothrombin time-international normalized ratio; APTT, activated partial thromboplastin time; Fib, fibrinogen; FDP, fibrin degradation product

Parameters	Patient Value	Reference Range
Serology		
CRP (mg/dL)	4.06	≤ 0.14
Lupus Anticoagulant (index)	121	≤ 1.2
aCL-Ab IgM (U/mL)	4.8	≤ 20
aCL-Ab IgG (U/mL)	5.0	≤ 20
aCL-β2GPI Ab IgG (U/mL)	9.3	≤ 20
aCL-β2GPI Ab IgM (U/mL)	1.3	≤ 20
Coagulation		
PT (sec)	68.1	11.0-13.4
PT (%)	7.2	≥ 74.0
PT-INR	6.19	0.94-1.15
APTT (sec)	283.7	24.0-39.0
Fib (mg/dL)	512	200-400
FDP (μg/mL)	3.2	≤ 5.0

**Figure 3:**
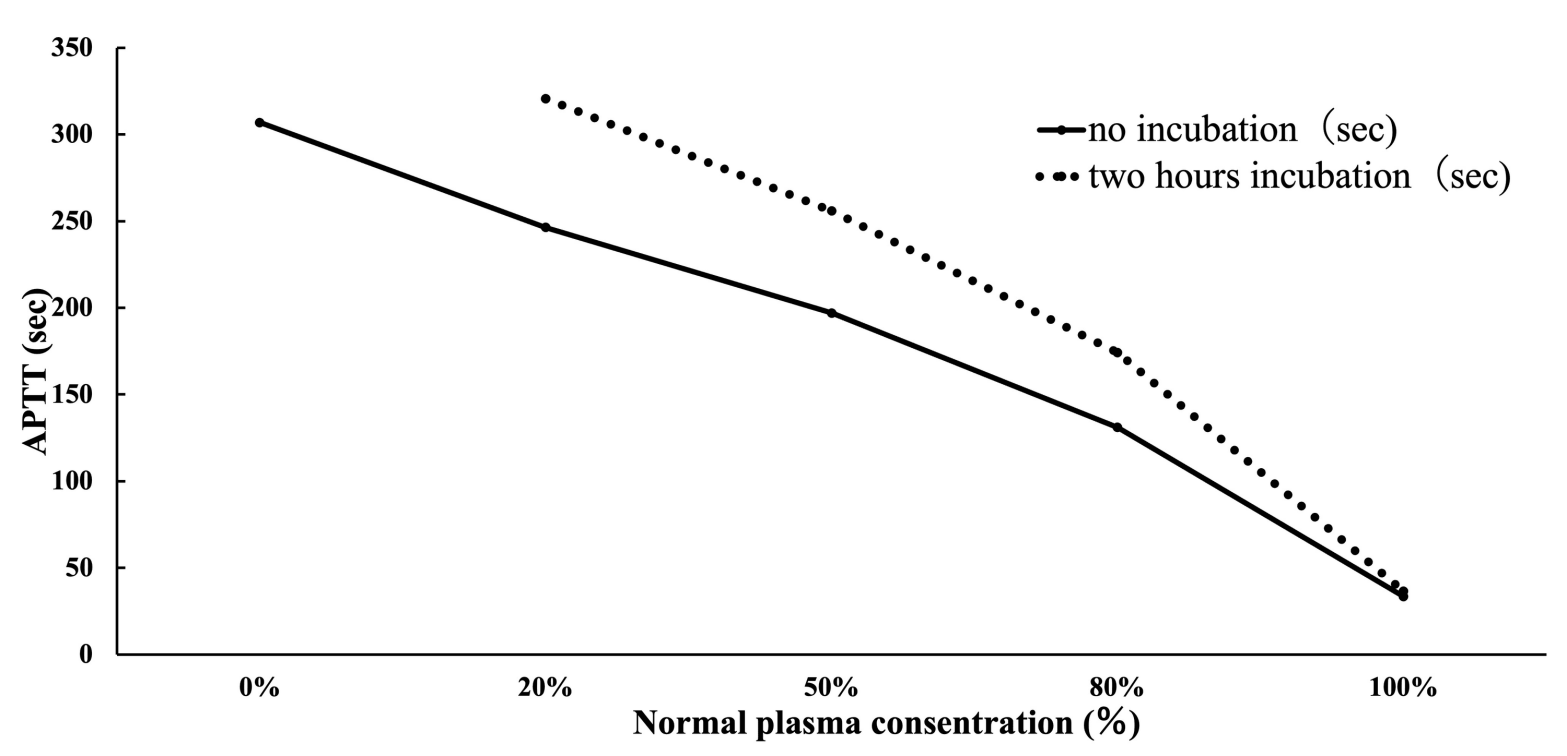
Result of an APTT cross-mixing test using normal stocked plasma. The solid line indicates the immediate reaction (no incubation), while the dotted line indicates the reaction after two hours of incubation. It showed an upward convex inhibitor pattern, suggesting the presence of inhibitors.

To differentiate whether the prolonged APTT was due to coagulation factor deficiency or the presence of an inhibitor in the coagulation system, an APTT cross-mixing test (CMT) was performed. Initially, APTT showed a linear pattern upon mixing with normal plasma, making interpretation difficult. On the other hand, after incubation at 37℃ for two hours, despite an increase in the proportion of normal plasma, the improvement in APTT was gradual, leading to the conclusion of an inhibitor pattern (Figure 3). Additionally, FV activity was found to be decreased to less than 3%. These findings led to the diagnosis of AiFVD, according to the diagnostic criteria established by the Japanese Ministry of Health, Labor, and Welfare [2].

**Figure 4:**
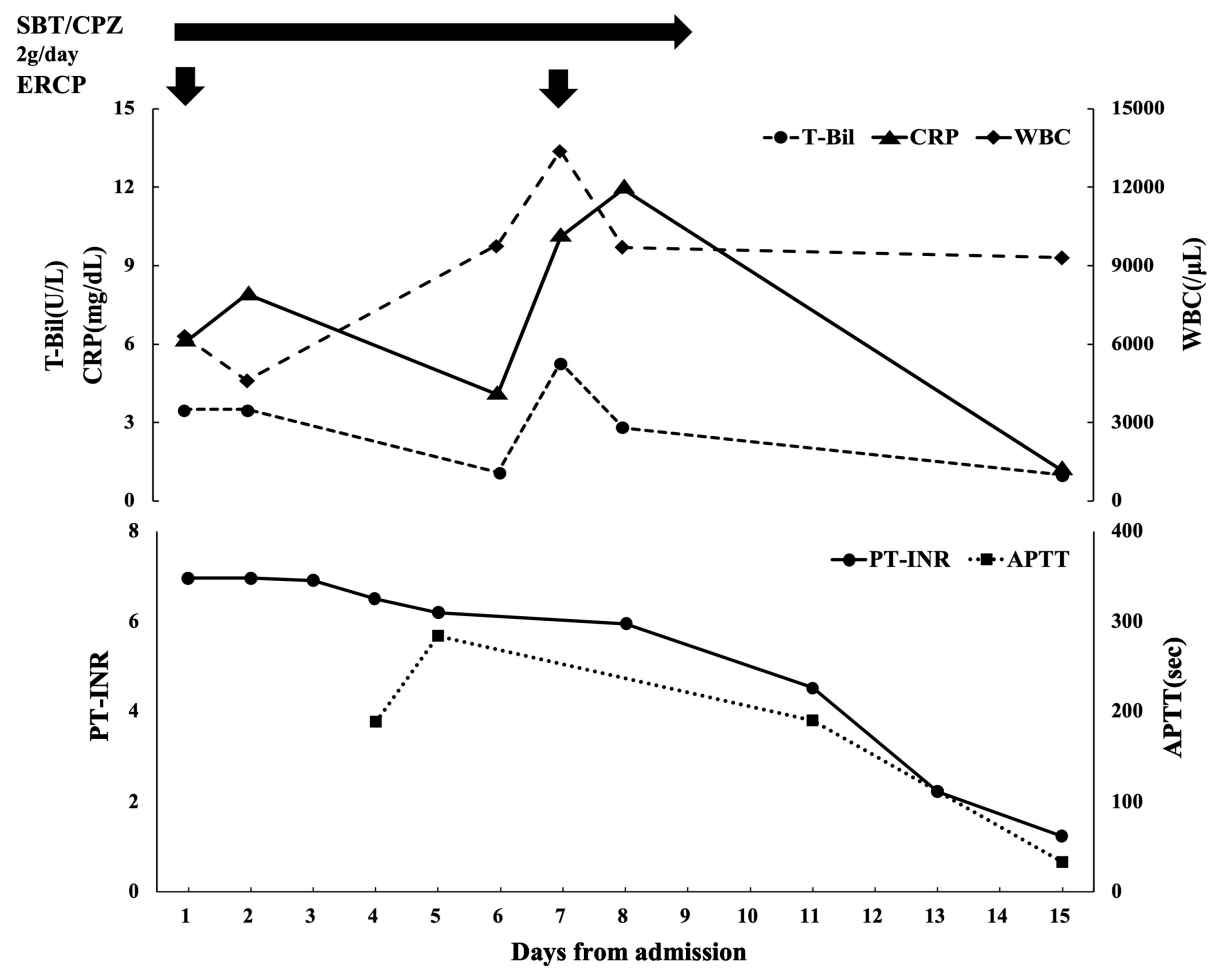
Clinical course. Horizontal axis: number of hospital days. Vertical axis: T-Bil and WBC, CRP, APTT, PT-INR. T-Bil, Total bilirubin, WBC: White blood cell, CRP: C-reactive protein, APTT: activated partial thromboplastin time (in seconds), PT-INR: international normalized ratio of prothrombin time, SBT/CPZ: Sulbactam/Cefoperazone, ERCP: endoscopic retrograde cholangiopancreatography.

Cholangitis persisted after the bile duct stent replacement. On the seventh day of hospitalization, the patient developed a recurrent fever, and laboratory findings showed elevated inflammatory markers and an increase in serum total bilirubin (T-Bil) and the inflammatory markers, including white blood cell count (WBC) and C-reactive protein (CRP), raising suspicion of recurrent cholangitis (Figure 4). Given the clinical deterioration, we decided to perform ERCP again. Dirty, purulent bile was drained from the papilla, and bile cultures confirmed infection, indicating that the initial ERCP had not achieved sufficient biliary drainage. Eight days after the second ERCP, serum T-Bil, WBC, and CRP had normalized (Figure 4). Coagulation tests, including FV activity, also showed improvement. The patient was able to resume chemotherapy after a 29-day interruption.

## Discussion

There are several coagulative disorders caused by a decrease in coagulation factors, such as vitamin K deficiency, liver disease, AHA, acquired von Willebrand syndrome, and AiFVD. AiFVD is an acquired bleeding disorder caused by a decrease in FV, leading to prolonged PT and APTT [2]. Although AiFVD may be asymptomatic, as in the present case, it can result in fatal hemorrhage. Therefore, early suspicion and accurate testing are essential for diagnosis and management [4,5].

Two systematic reviews of FV deficiency cases reported that bleeding symptoms occurred in 68.4% and 80.8% of patients, respectively [4,5]. A review on acquired FV inhibitors reported that bleeding symptoms, including hematuria, gastrointestinal bleeding, hematoma, intracranial hemorrhage, and pulmonary hemorrhage, were observed in patients, while, notably, 23% of patients were asymptomatic [8]. FV is a single-chain glycoprotein with a molecular weight of 330 kDa [9]. It is a vitamin K-independent coagulation factor belonging to the common pathway of the coagulation cascade. FV acts as a coenzyme that facilitates the activation of prothrombin to thrombin by the prothrombinase complex, with factor Xa playing a critical role [10]. It is predominantly synthesized in the liver, with approximately 80％ circulating in the blood and 20% in platelet α-granules [11]. The presence of FV in platelet α-granules plays a crucial role in the varied manifestations of AiFVD. FV in platelet α-granules is resistant to the neutralizing effects of inhibitors and is activated and released locally at the bleeding site after dissociation of multimerin-1 (MMRN1), a platelet protein that is present in endothelial cells and the extracellular matrix that is involved in hemostasis, coagulation, and cell adhesion. Therefore, bleeding symptoms are unlikely to occur if platelet counts are maintained. Furthermore, it has been reported that less than 1% of FV is sufficient for thrombin formation [11]. This likely explains why the patient in the present case did not experience bleeding symptoms, except for microscopic hematuria, despite FV activity being less than 3%.

The causes of AiFVD have been reported to include malignant tumors such as pancreatic cancer, infectious diseases, autoimmune diseases, and the administration of antibiotics, while approximately half of the cases remain idiopathic [12]. Malignant tumors may lead to the development of FV inhibitors, often associated with the presence of antiphospholipid antibodies (aPL) as a part of paraneoplastic syndrome, which may contribute to the development of AiFVD [13,14]. In the present case, aPL was also found to be positive; however, it is well recognized that the evaluation of aPL may be unreliable in the presence of significant coagulation abnormalities [13]. Although repeat testing after a 12-week interval is recommended to confirm a negative result [2], this was not performed in the current case. Therefore, although the involvement of aPL in the present case cannot be completely ruled out, the results of the CMT suggest that APS is unlikely. In cases of APS, the CMT typically demonstrates an upward convex or linear pattern in the immediate reaction, with no significant change after incubation at 37°C for two hours [15]. In contrast, the reaction in the present case exhibited a markedly enhanced upward convex pattern following two hours of incubation, which is indicative of the presence of an inhibitor. In autoimmune factor VIII deficiency (AiFVIIID) and AiFXIIID, autoimmune and infectious diseases have been reported to disrupt immune and inflammatory responses, contributing to its onset [12]. Although the precise mechanism underlying FV deficiency remains unclear, coagulation abnormality in AiFVD may arise through similar immunological pathways. In the present case, the coagulation abnormalities improved without any modification to the oncological management. Given the absence of temporal and clinical correlation between the progression of pancreatic cancer and the coagulation disorder, it is unlikely that pancreatic cancer was the underlying cause of AiFVD. In contrast, the resolution of the coagulation abnormalities following treatment for cholangitis suggests that infection was the most probable trigger for AiFVD in this case.

Thrombocytopenia is occasionally observed in AiFVD, and the coexistence of thrombocytopenia and coagulation abnormalities necessitates careful differentiation from disseminated intravascular coagulation (DIC). Cholangitis is a known precipitating factor for DIC, in which anticoagulant therapy may be required. However, in AiFVD with thrombocytopenia, such treatment may exacerbate hemorrhagic risk, underscoring the importance of accurate differential diagnosis. In cases of infection associated with coagulopathy and thrombocytopenia, clinicians should be cautious and consider AiFVD as a potential diagnosis, rather than hastily attributing the symptoms to DIC. While elevated thrombin-antithrombin complex (TAT) levels are a hallmark of DIC, such increases are typically absent in AiFVD, potentially aiding in the differential diagnosis. The key clinical and laboratory differences between AiFVD and DIC are summarized in Table 4. In the present case, although TAT levels were not measured, the diagnosis of DIC was considered unlikely based on the presence of a normal platelet count and elevated plasma fibrinogen levels [16]. 

**Table 4:** The key clinical and laboratory differences between AiFVD and DIC AiFVD, autoimmune acquired factor V deficiency; DIC, disseminated intravascular coagulation; FDP, fibrin/fibrinogen degradation products

Parameter	AiFVD	DIC
Platelet count	Usually normal	Decreased
PT / APTT	Both markedly prolonged	Mild-to-moderate prolongation
Fibrinogen	Normal or elevated	Decreased
D-dimer / FDP	Usually normal	Elevated
Factor V activity	Markedly decreased	Slightly decreased (due to consumption)
TAT (thrombin-antithrombin complex)	Typically normal (not measured in our case)	Markedly elevated
Bleeding tendency	Variable(it may be absent)	Present in advanced cases

The primary therapeutic approach for AiFVD involves the control of bleeding and the elimination of circulating inhibitors. Currently, no FV concentrates are commercially available for replacement therapy [5]. In cases of active hemorrhage, the administration of fresh frozen plasma (FFP) and platelet transfusions is recommended. Notably, platelet transfusions may be more effective than FFP in certain cases, given that FV stored in platelet α-granules is believed to play a more critical role in hemostasis than plasma-derived FV [17]. Alternative treatment options include the use of activated prothrombin complex concentrate and recombinant activated factor VII (FVII) [17,18]. FVII can activate FX via the extrinsic coagulation pathway, thereby compensating for reduced FV activity and promoting thrombin generation and fibrin clot formation-both essential processes for effective hemostasis [19,20].

Various strategies have been reported for the reduction or removal of FV inhibitors, including corticosteroid therapy, plasma exchange, immunoadsorption, and intravenous immunoglobulin (IVIg) [6]. In the present case, however, none of these interventions were employed, as the patient exhibited no overt bleeding symptoms apart from microscopic hematuria. Coagulation abnormalities resolved solely with treatment of the underlying cholangitis. It is noteworthy that spontaneous resolution of FV inhibitors has been reported in approximately 10.5% of AiFVD cases [2]. On the other hand, recurrence has been documented in approximately 11% of patients [14]. Therefore, even in cases with mild or spontaneously resolving symptoms, as demonstrated in our patient, long-term follow-up remains essential in the management of AiFVD.

## Conclusions

AiFVD is a rare and complex coagulation disorder that can manifest with a spectrum of clinical presentations, ranging from asymptomatic cases to severe hemorrhage. In this report, we describe a case of AiFVD triggered by cholangitis, wherein coagulation abnormalities improved following treatment of the underlying cholangitis alone. These findings suggest that inflammatory conditions, such as cholangitis, may contribute to the development of FV inhibitors. Even in the absence of overt bleeding, clinicians should consider AiFVD in patients presenting with unexplained coagulation abnormalities. Understanding potential triggers is crucial for timely diagnosis, especially in patients with no prior history of bleeding disorders. Early recognition and appropriate management of the underlying cause may allow for spontaneous resolution without the need for immunosuppressive therapy.

## References

[1] Collins PW, Hirsch S, Baglin TP (2007). Acquired hemophilia A in the United Kingdom: a 2-year national surveillance study by the United Kingdom Haemophilia Centre Doctors' Organisation. Blood.

[2] Ichinose A, Osaki T, Souri M (2022). A review of coagulation abnormalities of autoimmune acquired factor V deficiency with a focus on Japan. Semin Thromb Hemost.

[3] Ichinose A, Osaki T, Souri M (2025). Diagnosis and treatment of autoimmune acquired coagulation factor deficiencies: an evidence-based review of Japanese practice. Semin Thromb Hemost.

[4] Ang AL, Kuperan P, Ng CH, Ng HJ (2009). Acquired factor V inhibitor. A problem-based systematic review. Thromb Haemost.

[5] Franchini M, Lippi G (2011). Acquired factor V inhibitors: a systematic review. J Thromb Thrombolysis.

[6] Godart B, Boinot C, Remblier C, Hajjar A, Beauchant M (2006). Acquired factor V inhibitor associated with valproic acid use in a cirrhotic patient. Gut.

[7] Knöbl P, Lechner K (1998). Acquired factor V inhibitors. Baillieres Clin Haematol.

[8] Miura F, Okamoto K, Takada T (2018). Tokyo Guidelines 2018: initial management of acute biliary infection and flowchart for acute cholangitis. J Hepatobiliary Pancreat Sci.

[9] Boland F, Shreenivas AV (2017). Acquired factor V inhibitors: a review of the literature. Ann Hematol Oncol.

[10] Tracy PB, Eide LL, Bowie EJ, Mann KG (1982). Radioimmunoassay of factor V in human plasma and platelets. Blood.

[11] Donohoe K, Levine R (2015). Acquired factor V inhibitor after exposure to topical human thrombin related to an otorhinolaryngological procedure. J Thromb Haemost.

[12] Duckers C, Simioni P, Rosing J, Castoldi E (2009). Advances in understanding the bleeding diathesis in factor V deficiency. Br J Haematol.

[13] Favaloro EJ, Posen J, Ramakrishna R (2004). Factor V inhibitors: rare or not so uncommon? A multi-laboratory investigation. Blood Coagul Fibrinolysis.

[14] Bayani N, Rugina M, Haddad-Vergnes L, Lelong F (2002). High-titer acquired factor V inhibitor responsive to corticosteroids and cyclophosphamide in a patient with two malignant tumors. Am J Hematol.

[15] Kumano O, Moore GW (2019). Ruling out lupus anticoagulants with mixing test-specific cutoff assessment and the index of circulating anticoagulant. Res Pract Thromb Haemost.

[16] Vetri D, Lumera G, Tarascio S, Scuto S, Marino E, Barcellona G, Signorelli SS (2020). A case of acquired factor V deficiency in patient with bleeding. TH Open.

[17] Chediak J, Ashenhurst JB, Garlick I, Desser RK (1980). Successful management of bleeding in a patient with factor V inhibitor by platelet transfusions. Blood.

[18] William BM (2008). Adjunctive role for recombinant activated factor VII in the treatment of bleeding secondary to a factor V inhibitor. Blood Coagul Fibrinolysis.

[19] Franchini M, Lippi G (2010). Recombinant activated factor VII: mechanisms of action and current indications. Semin Thromb Hemost.

[20] Lippi G, Favaloro EJ, Montagnana M, Manzato F, Guidi GC, Franchini M (2011). Inherited and acquired factor V deficiency. Blood Coagul Fibrinolysis.

